# Tensile Behavior of High-Density Polyethylene Including the Effects of Processing Technique, Thickness, Temperature, and Strain Rate

**DOI:** 10.3390/polym12091857

**Published:** 2020-08-19

**Authors:** Mohammad Amjadi, Ali Fatemi

**Affiliations:** Mechanical Engineering, University of Memphis, Memphis, TN 38152, USA; M.Amjadi@memphis.edu

**Keywords:** tensile behavior, HDPE, temperature effect, strain rate effect, processing technique effect

## Abstract

The primary goal of this study was to investigate the monotonic tensile behavior of high-density polyethylene (HDPE) in its virgin, regrind, and laminated forms. HDPE is the most commonly used polymer in many industries. A variety of tensile tests were performed using plate-type specimens made of rectangular plaques. Several factors can affect the tensile behavior such as thickness, processing technique, temperature, and strain rate. Testing temperatures were chosen at −40, 23 (room temperature, RT), 53, and 82 °C to investigate temperature effect. Tensile properties, including elastic modulus, yield strength, and ultimate tensile strength, were obtained for all conditions. Tensile properties significantly reduced by increasing temperature while elastic modulus and ultimate tensile strength linearly increased at higher strain rates. A significant effect of thickness on tensile properties was observed for injection molding specimens at 23 °C, but no thickness effect was observed for compression molded specimens at either 23 or 82 °C. The aforementioned effects and discussion of their influence on tensile properties are presented in this paper. Polynomial relations for tensile properties, including elastic modulus, yield strength, and ultimate tensile strength, were developed as functions of temperature and strain rate. Such relations can be used to estimate tensile properties of HDPE as a function of temperature and/or strain rate for application in designing parts with this material.

## 1. Introduction

Application of polymeric materials and their composites have been increasing rapidly in different industries, such as the automotive industry, due to their advantages such as lighter weight and resistant to corrosive environments, as compared to metals [1,2,3]. Polyethylene is the world’s most widely used polymer in volume. Compared to other polymers, polyethylene has outstanding characteristics such as toughness, abrasion resistance, impact resistance, low (near zero) water absorption, low cost, and recyclability.

There are three major grades of polyethylene; low density, medium density and high-density polyethylene (LDPE, MDPE, and HDPE), depending on molecular density and crystallinity of the polyethylene structure. High Density Polyethylene (HDPE) has high rigidity, strength, and better creep behavior. Global demand for High-Density Polyethylene (HDPE) resins has been increasing, going from 11.9 million tons in 1990 to 43.9 million tons in 2017 with an annual growth of 3.3% [4]. 

Molecular constitution and microstructural aspects like the degree of crystallinity, crystal size, crystal thickness, and crystal orientation may affect the physical and mechanical properties of polyethylene [5,6,7,8,9,10]. It is a fast crystallizing polymer at elevated temperatures where the thermally activated crystallization becomes significant [5]. The amount of crystallinity affects the mechanical properties of semi-crystalline polymers such as HDPE. Mechanical properties and morphological changes in HDPE also significantly depend on the orientation of the deformations with respect to the molecular and crystal arrangement [6]. Yield stress and lamella thickness are proportional to crystallinity and increase linearly with the index of crystallinity [7].

Based on experimental observations, while the ultimate tensile strength depends on the crystallinity level, it is independent of molecular weight [9]. However, Karasev et al. [8] observed that tensile strength depends on the molecular weight distribution at elevated temperatures (80–100 °C). On the other hand, the yield point below about −100 °C is insensitive to the structure and is dependent on factors such as the strength of the van der Waals’ bonds, but not on the details of the morphology [10].

High molecular weight results in higher strength due to the low capability of sliding molecules over each other. In addition, increasing molecular weight reduces crystallinity [11]. Crystallinity and secondary bond strength control the stiffness of thermoplastic, while intra-chain, inter-chain, secondary bonding and crystallinity govern the strength of thermoplastics. As mentioned, the degree of crystallinity plays a more important role on HDPE mechanical properties than molecular weight [11,12]. Processing conditions can also influence the microstructure parameters and consequently mechanical properties [13]. Temperature gradient during the manufacturing process may affect crystallinity and higher mold temperature leads to a lower crystallization rate and improved modulus [14].

HDPE can be manufactured either as virgin or regrind (recycled) material. Regrind HDPE is the excess material form production line (trimming or cutoffs), which is used again in the production line to reduce material waste. Regrind HDPE material experiences more than one thermomechanical history as compared to virgin material. The physical and mechanical properties of regrind material are often not published because of too many variables involved [11].

Polyethylene has the ability to permeate chemical liquids, gas, and vapors which is not desired in some applications such as packaging, chemical storage containers, and automotive fuel tanks. Therefore, a barrier layer is usually introduced in a coextruded multilayer structure to minimize permeability of polyethylene. For example, in automotive fuel tanks made of multilayered HDPE structure, a barrier layer ranging from 2 to 5% of the total thickness is used to reduce the gasoline permeability [15].

The most common processing techniques to manufacture HDPE are extrusion, injection molding, blow molding, and compression molding. Manufacturing techniques may influence the mechanical response of HDPE, such as elastic modulus and tensile strength, which could be due to different molecular morphologies and structures in the final product [16]. It has been shown that the optimization of manufacturing parameters in injection molding results in substantially improved tensile properties [17].

Tensile testing is the most common mechanical testing performed on different materials due to the simplicity and low cost and due to the fact that tensile properties are still widely used in design. In addition, tensile properties can be used in semi-empirical models for creep and/or fatigue analysis. Stiffness, yield strength, ultimate tensile strength, and toughness are the basic tensile properties which can be characterized by tension tests.

Zhou and Wilkes [6] investigated the orientation effect on mechanical and morphological properties for uniaxially melt-extruded HDPE films. It was shown that tensile properties were significantly dependent on the orientation of molecular structure originating from different morphologies developed during the polymer deformation process [6]. Anisotropy effect in tensile properties was also investigated through tension tests in 0°, 45°, and 90° relative to extrusion direction for two grades of polyethylene (LDPE and UHMWPE) [18]. No significant difference on tensile properties was observed among samples and it was concluded that the extruded polyethylene material is isotropic in terms of tensile behavior [18]. However, Grommes et al. [19] reported significant anisotropy effect in elastic modulus for blow molded HDPE between extrusion direction and blowing direction (perpendicular to extrusion) for blow molded HDPE. Elastic modulus in the direction of extrusion was approximately 8% higher than in the direction perpendicular to it. [19].

Schrauwen [13] studied flow-induced oriented structures in injection molded HDPE samples. Tensile tests were performed along and perpendicular to the injecting direction. Tensile yield strength was higher in the flow direction due to the amount of oriented extended chain crystals. It was also observed that the larger the thickness the lower the amount of orientation.

Addiego et al. [7] studied the manufacturing technique effect on tensile properties of HDPE. Tension samples were cut from extruded (7 mm thickness), injection molded (4 mm thickness), and compression molded (6 mm thickness) plates. Tensile tests were carried out at 40 °C under a constant true strain rate of 5 × 10^−3^ s^−1^. Microstructural characterization was also performed to measure and compare the crystallinity and molecular weight of these three sets of samples with different manufacturing techniques. They showed that ultimate tensile strength linearly increases with increasing crystallinity.

Tensile behavior can change from a ductile to a brittle manner at different loading rates or service temperatures [20,21,22]. Strain rate has a significant effect on tensile properties due to the semi-crystalline nature of HDPE and the contribution of viscosity in the mechanical response under monotonic deformation. At high strain rates, molecular chains are unable to coordinate and deform as fast as the load is applied. It has been observed that elastic modulus and ultimate tensile strength increase at higher strain rates [23,24,25,26,27].

Dasari et al. [28] evaluated tensile behavior of injection molded HDPE (density = 0.95 g/cm^3^, crystallinity = 67%) at room temperature and three strain rates 0.016, 0.04, and 0.08 s^−1^. A linear relationship in a semi-log plot was observed between the ultimate tensile stress and strain rate. SEM imaging from fractured surfaces showed that at low strain rates polyethylene behaves in a ductile manner and fracture is a result of polymer chain fibrillation. At intermediate strain rates, crazing or tearing was the predominant mode of fracture at the edges, while fibrillar failure occurred in the mid-thickness region. At high strain rates, the percentage of fibrillation was small compared to that at low strain rates. Addiego et al. [29] also found ultimate tensile stress (true stress) linearly increased with log of true strain rate for HDPE (density = 0.962 g/cm^3^, crystallinity = 78%) at 23 °C.

Merah et al. [30] investigated the effect of temperature on tensile properties of HDPE (PE-100 pipe grade HDPE with density = 0.96 g/cm^3^ ) at a constant engineering strain rate of 0.0006 s^−1^. A significant reduction in ultimate tensile strength and elastic modulus was observed by increasing temperature from −10 to 70 °C. Ductile fracture surface was observed even at the cold temperature of −10 °C). 

Effects of temperature and strain rate on tensile properties were investigated for compression molded HDPE at −40, 23, and 70 °C in strain rate range of 0.01–10 s^−1^ in [31]. Again, by increasing strain rate, elastic modulus and ultimate tensile strength increased linearly at each tested temperature. The trend of reduction of tensile properties with increasing temperature was also found to be linear [12]. X-ray diffraction images from the stretched polyethylene films showed that elastic modulus of polyethylene crystals is nearly independent of temperature up to 145 °C, while Young’s modulus (*E*) of the films decreased by increasing temperature [13].

Based on the literature review, tensile properties could significantly depend on the orientation of molecular structure originating from different morphologies developed during the polymer deformation process. Tensile properties also significantly change with the degree of crystallinity. Strain rate and/or operating temperature can change the fracture mode from ductile to brittle and have a substantial influence on tensile properties. Different relations have been developed in the literature to relate tensile properties to strain rate or temperature. 

In this paper, the effects of manufacturing technique, thickness, temperature, and strain rate on tensile properties of virgin, regrind, and laminated HDPE are investigated. The obtained experimental data were used to develop empirical relationships as functions of both strain rate and temperature for tensile properties. The experimental program conducted as well as the results obtained are presented and discussed in the following sections.

## 2. Materials and Methods 

High-density polyethylene as virgin, regrind, and laminated HDPE was studied with three processing techniques (injection molding, blow molding, and compression molding) and with three specimen thicknesses of 1, 2 and 4 mm. Virgin HDPE specimens with 0.2 % wt of carbon black were also tested to investigate the effect of carbon black addition on tensile properties. Flash is produced during the blow molding process; for example, 70 percent for an automotive duct [15]. Up to 25% regrind HDPE may be used for manufacturing multilayer HDPE fuel tanks in the automotive industry [15]. Therefore, regrind and laminated HDPE forms were also included in the experimental study. Laminated HDPE is a multilayer composite of virgin and regrind forms with a very thin layer of adhesive and barrier (about 3% of the total thickness). The main layers are virgin HDPE, regrind HDPE, a barrier layer as Ethylene vinyl alcohol (EVOH), and an adhesive layer (LDPE).

**Figure 1:**
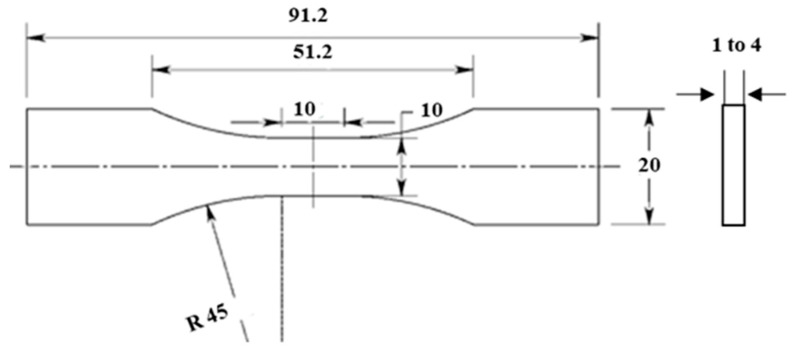
Flat specimen geometry used for tension tests. Dimensions are in mm.

Virgin and regrind HDPE specimens were machined from compression-molded rectangular plates with dimensions of 170 × 190 mm^2^ and thicknesses of 1, 2 and 4 mm. For blow molded HDPE, rectangular plates with dimensions of 160 × 160 mm^2^ and thickness of 4 mm were used to make test specimens. To study any anisotropy effect, compression molded and injection molded specimens were cut in both transverse and longitudinal directions. Finally, laminated specimens were cut and prepared from blow molded multilayer polyethylene plates. All the test specimens were machined from strips cut from plates using a CNC milling machine with the geometry shown in Figure 1.

Tension tests were performed in displacement-controlled mode according to ASTM D638 [32] and ISO-527 [33] test standards using an 8800 servo-hydraulic testing machine (Instron, Norwood, MA, US). Tensile strain was measured using a non-contact video extensometer (Instron, Norwood, MA, US). Two dots in the consistent distance of 8 mm in the gage section of specimens were marked. A video extensometer was used to measure the distance between these two points and the resultant strain as the specimen elongated. An environmental chamber (Instron, Norwood, MA, US) equipped with an electronic heating element and a liquid nitrogen cooling system (accuracy of ±1 °C) was used for conducting temperature effect tests. 

Tension tests were conducted at four temperatures (−40, 23, 53 and 82 °C) and three load actuator speeds (1, 10, and 100 mm/min) for virgin HDPE (compression molded) and laminated HDPE (blow molded) specimens to investigate the effects of temperature and strain rate on tensile properties. Virgin and regrind forms with injection molding and blow molding processing techniques were tested only at 23 °C and one strain rate (0.0006 s^−1^).

Elastic modulus, 0.2% offset yield strength, and ultimate tensile strength were measured from the tension tests. The yield point is defined as the point at which deviation from linearity exceeds a specified offset value (0.2% in this case). However, sometimes in the polymer literature the ultimate tensile strength is called the yield strength. Ultimate tensile strength (*S_u_*) is usually reported as the maximum engineering stress (maximum force divided by the original cross-sectional area of the specimen) measured during a tensile test. Ultimate tensile strength happens at the onset of necking in the case of ductile material behavior.

## 3. Results and Discussion 

### 3.1. Manufacturing Process Effect

Mechanical properties of polymers may change in different directions because of processing conditions due to arrangements or molecular orientations along molding direction. Polymer chains can be arranged parallel and perpendicular to the primary direction of resin flow. This biaxial orientation of polymer chains can be achieved in blow molding or blown-film extrusion [11].

As mentioned earlier, virgin HDPE specimens (injection molded and compression molded with 4 mm thickness) were tested in two perpendicular directions. No anisotropy effect was observed for virgin HDPE with compression molding. Isotropy was expected since the melted resin in this case is exposed to multi-direction flow, leading to the same molecule orientation in all directions.

However, tensile properties were slightly different between two perpendicular directions for virgin HDPE with the injection molding processing technique. The maximum difference of the average tensile strength and elastic modulus were 0.4 (~2%) and 185 MPa (~19%) between the directions along and perpendicular to melt flow direction, respectively. During injection molding, the induced shear to the melted polymer might cause aligning of the molecular chains. This mechanical deformation creates a structure regularity along the flow direction. 

Polarized light microscopy has also revealed that the compression molded HDPE structure is more homogeneous than the injection molded HDPE [34]. In addition, as melt flow cools down, the orientation of molecules in flow direction is frozen. Therefore, tensile strength in the flow direction may be higher than the cross-flow direction [35]. Mold temperature, part thickness, and flow thickness can affect the orientation of polymer molecules in injection molding.

**Figure 2:**
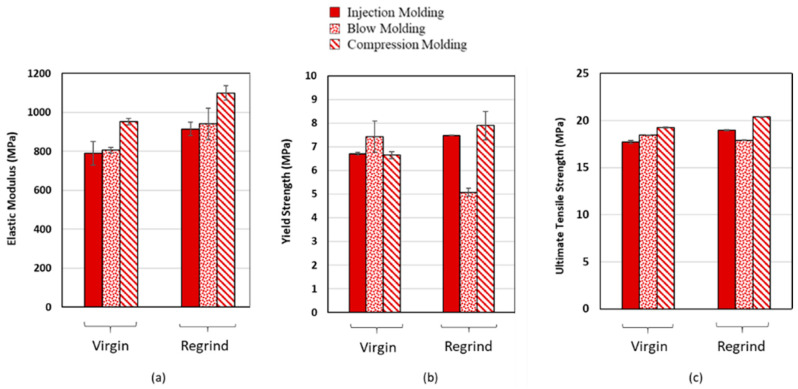
(**a**) Elastic modulus, (**b**) yield strength, and (**c**) ultimate tensile strength for virgin and regrind HDPE, with three processing techniques at RT and 6 × 10^−4^ s^−1^ strain rate. Results shown are for average of two tests in each condition.

Figure 2a–c show elastic modulus, yield strength, and ultimate tensile strength, respectively, for two materials (virgin and regrind) with the same thickness (4 mm), and with three processing techniques. As can be seen, there are some improvements by the regrinding process in elastic modulus and ultimate tensile strength, as compared to virgin HDPE. The improvement for the regrind may be attributed to the more than one thermal process history on the HDPE resin which can break polyethylene molecular chains and align them more along the flow direction. However, no difference in tensile properties between virgin and regrind HDPE at RT or at −40 °C was reported in [11]. Figure 2 also shows compression molding results in higher tensile properties as compared to injection molding and blow molding for both virgin and regrind HDPE. However, tensile properties of both materials are similar for blow molding and injection molding.

**Figure 3:**
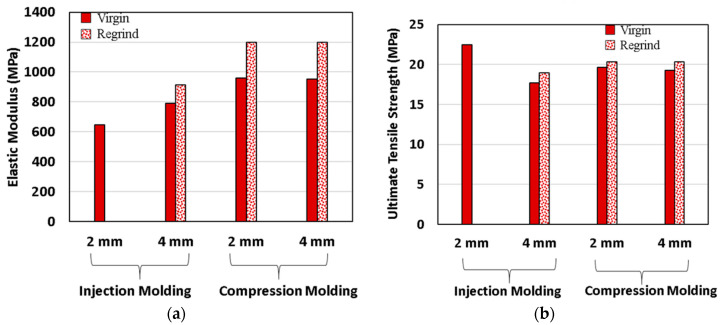
(**a**) Elastic modulus and, (**b**) ultimate tensile strength comparison due to thickness effect for virgin and regrind HDPE at room temperature (RT) and 6 × 10^−4^ s^−1^ strain rate.

Elastic modulus and ultimate tensile strength for virgin and regrind HDPE with 2 and 4 mm thicknesses are shown in Figure 3a,b, respectively. These properties are not significantly different due to thickness effect in compression molding process, either for virgin or regrind HDPE. However, there is a significant thickness effect on elastic modulus and ultimate tensile strength for the injection molding processing technique. The elastic modulus of specimens with 4 mm thickness was 23% higher than for 2 mm thickness. On the other hand, the ultimate tensile strength of specimens with 4 mm thickness was about 21% lower than 2 mm thickness. The reason for higher ultimate tensile strength for 2 mm specimens may be attributed to the existence of surface layers with higher molecular orientation, whereas in specimens with 4 mm thickness molecular orientation is decreased from layers closer to the surface, as compared to layers closer to the core region.

### 3.2. Temperature Effect

**Figure 4:**
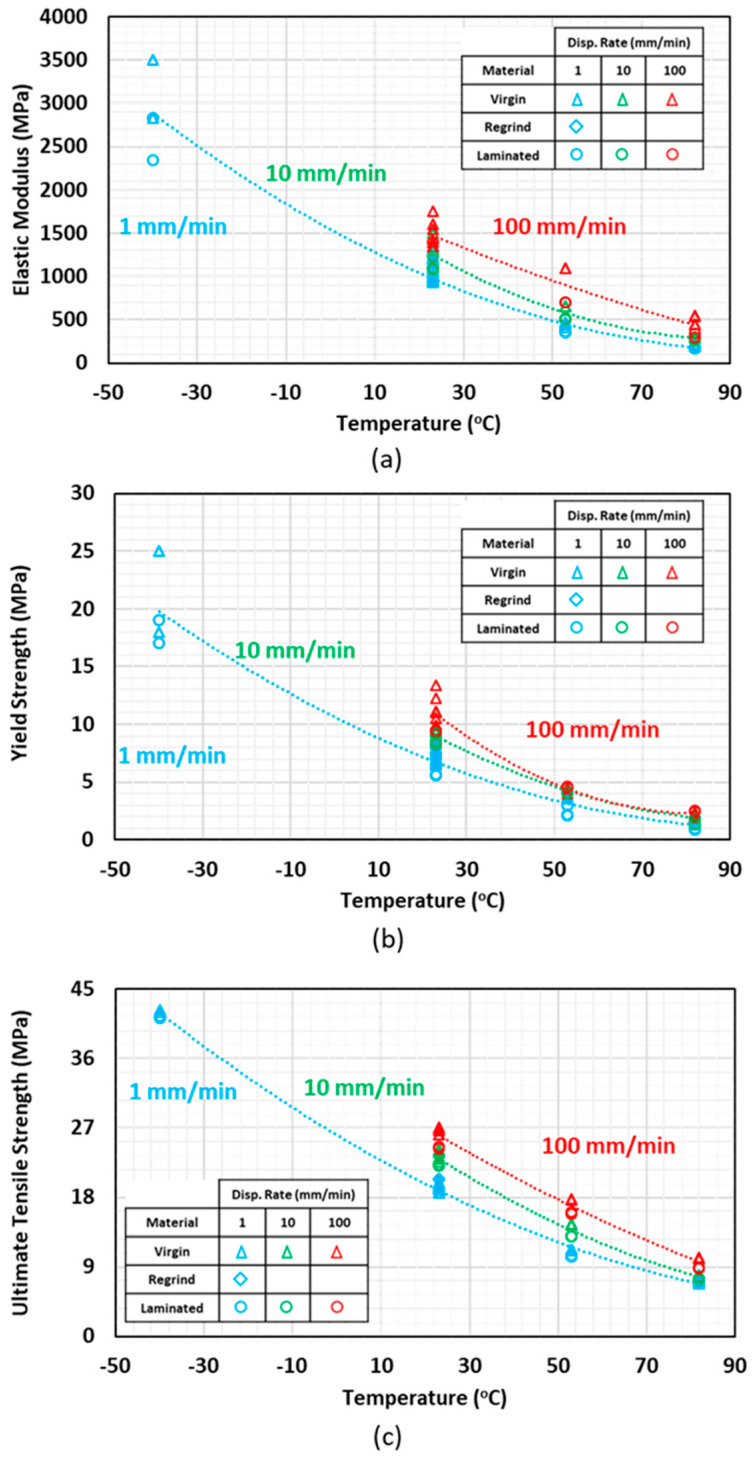
(**a**) Elastic modulus, (**b**) yield strength, and (**c**) ultimate tensile strength variations with temperature at three different strain rates.

Temperature plays an important role in mechanical properties of polymers. Variations of *E*, *S_y_*, and *S_u_* with temperature are shown in Figure 4a–c respectively. Virgin, regrind, and laminated HDPE with compression and blow molding processing techniques are included in the plots. Necking happened at all temperatures and strain rates and the specimens elongated continuously after reaching ultimate tensile strength, even at the slowest tested strain rate and temperature of −40 °C.

HDPE shows higher stiffness and ultimate tensile strength at low temperatures due to lower chain mobility than at elevated temperatures. At elevated temperatures, the molecules are more flexible and can deform in the direction in which stress is applied. Physical bonds due to Van der Waals hydrogen, or dipole–dipole interactions restrict molecular motions and define the initial stiffness. Due to low glass transition temperature of HDPE (−110 °C), it is in its rubbery phase at −40 °C. By increasing temperature, strain at ultimate tensile strength increases, indicating higher ductility.

As the test temperature increased, an exponential reduction in tensile properties was observed for all materials (virgin, regrind, and laminated) with different processing techniques. This reduction is more significant by increasing temperature from −40 to 23 °C, as compared to the reduction from 53 to 82 °C, as the temperature is getting closer to HDPE melt temperature (~130 °C). As temperature increases, secondary (physical) bonds between polymer chains in the amorphous phase break and tensile properties decline. Significant elongation at 82 °C is attributed to higher mobility and breakage of tie molecules in the amorphous phase. 

The reduction trends in tensile properties with temperature for virgin, regrind, and laminated HDPE are shown in Figure 4. The strain at ultimate tensile strength increases for both virgin and laminated HDPE specimens at 53 and 82 °C, as compared to at RT.

### 3.3. Strain Rate Effect

The strain rate at which specimens are deformed significantly affects their response to the applied stress. As the strain rate increases the molecular mobility of the polymer chains decreases and material becomes stiffer. Higher strain rate results in increased elastic modulus, higher tensile strength, and lower strain at ultimate tensile strength. Fracture mode can also vary from ductile to brittle depending on the strain rate. A ductile behaving material at low strain rate can exhibit brittle fracture at high strain rate.

**Figure 5:**
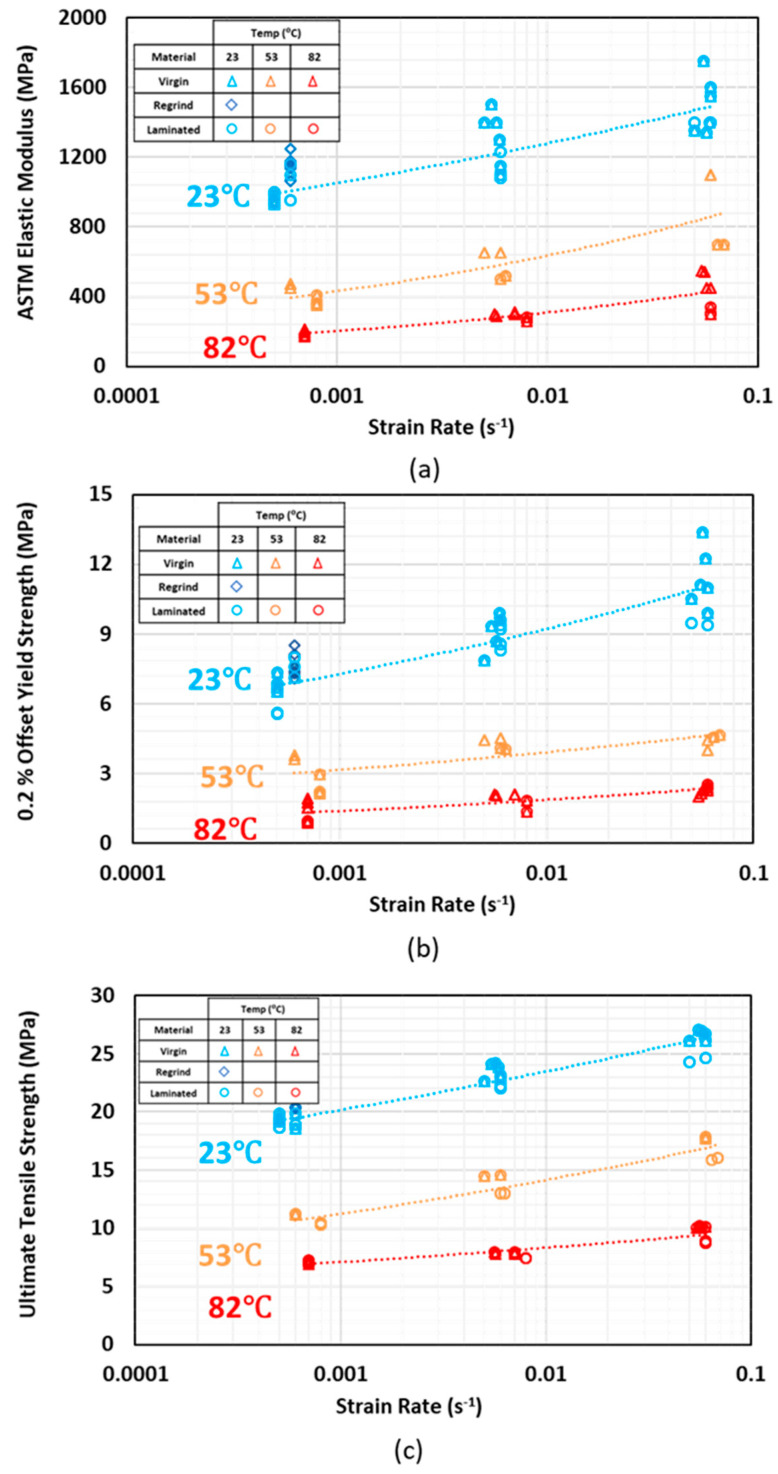
(**a**) Elastic modulus, (**b**) yield strength, and (**c**) ultimate tensile strength variations with strain rate at different temperatures.

Elastic modulus and ultimate tensile strength improved by increasing strain rate from 0.0006 to 0.006 and 0.06 s^−1^, as shown in Figure 5. In addition, strain at ultimate tensile strength reduced at higher strain rates, therefore, the material behavior becomes more brittle as strain rate increases. As can be seen from Figure 5, the slope of the lines at elevated temperatures is decreased, therefore, strain rate sensitivity of tensile properties is reduced by increasing temperature. This may be explained by breaking of tie molecules that connect the amorphous and crystalline phases. As the rate of loading increases, molecular chains cannot coordinate and deform, hence, material becomes more brittle and flexibility decreases.

**Figure 6:**
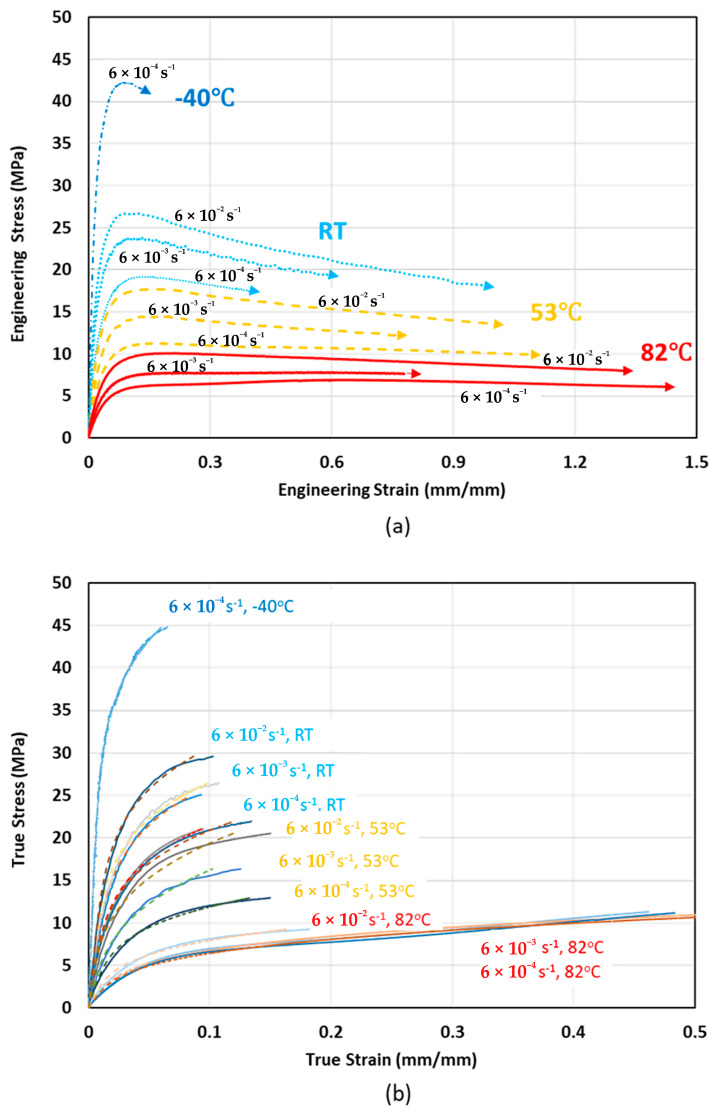
(**a**) Engineering stress-strain curves, and (**b**) true stress-strain curves showing temperature and strain rate effects at different temperatures and strain rates.

Since semi-crystalline polymers contain both crystalline and amorphous phases, if the strain rate is lower than lamella (crystalline phase), then the molecular chains cannot be disentangled and stiffness and strength increase. On the other hand, if the strain rate is low enough to let crystalline molecular chains be disentangled, then stiffness and strength reduce. As shown in Figure 6a, elastic modulus and ultimate tensile strength increase by increasing the strain rate at a constant temperature, while tensile properties reduce by increasing temperature at a constant strain rate. The effect of increasing deformation rate is similar to the effect of decreasing test temperature, as can be seen from Figure 6a.

A linear relationship was obtained between the actuator displacement rate and specimen gage section strain rate in the log-log scale, expressed as:

(1) $$\dot{\varepsilon }=a\;\dot{\delta }$$

where $\dot{\delta }$ is the displacement rate in mm/min, $\dot{\varepsilon }$ is strain rate in mm^−1^, and a is a constant obtained to be about 0.0351 mm^−1^ for the specimen geometry used for all the HDPE forms and at all temperatures tested.

### 3.4. Representation of Tensile Curves and an Emprical Model for Tensile Properties

Different mathematical models can be used to represent the monotonic stress-strain curve, which can be used in numerical simulations such as finite element analysis. The Ramberg-Osgood (RO) equation has been shown to represent true stress-strain curves of neat and short fiber reinforced polymers very well [20,22,36,37,38]. This equation was used to represent true stress-strain curves up to the ultimate tensile strength of the studied materials at all temperatures and strain rates and is expressed as:

(2) ε=σE+(σK)1n

(3) $$\sigma =K\;\varepsilon_{p}^{n}$$

where K is the strength coefficient and n is the strain hardening exponent. The constants K and n are obtained from a fit of true stress (σ) versus true plastic strain ($\varepsilon_{p}$) on a log-log scale [39]. Figure 6b shows true stress versus true strain and the superimposed experimental curve with the RO representation. 

A set of two-variable functions for tensile properties; elastic modulus (E), yield strength ($S_{y}$), and ultimate tensile strength ($S_{u}$) were developed using the obtained tensile properties at different temperatures (T) and strain rates ($\dot{\varepsilon }$). The fitted equations can be represented in Equation (4) given by:

(4) $$S_{y},\;S_{u},\;E=D+C\;\left(Log\dot{\varepsilon }\right)+B\;\left(T\right)+A\;\left(T^{2}\right)$$

where temperature T is in °C and strain rate $\dot{\varepsilon }$ is in s^−1^ and A, B, C, and D are empirical constants obtained from the data fits and listed in Table 1. 

**Figure 7:**
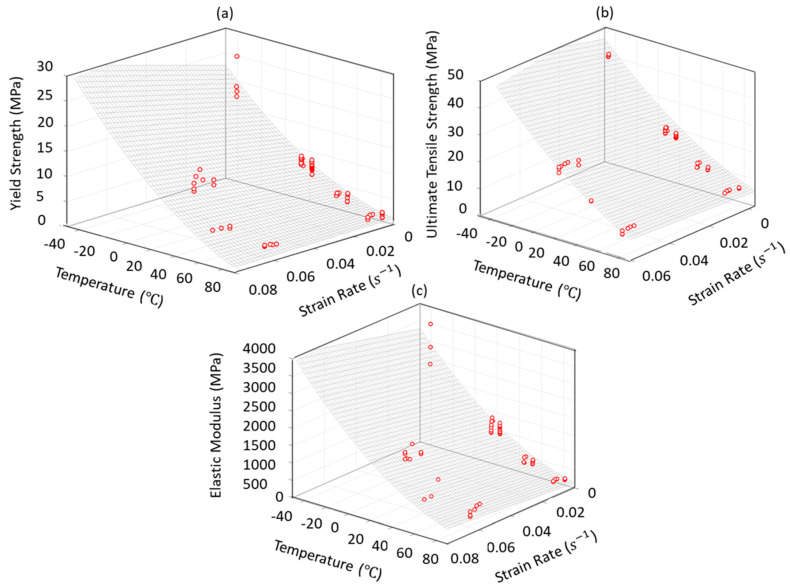
3D representation of yield strength (**a**), ultimate tensile strength (**b**), and elastic modulus (**c**) as polynomial functions of temperature and strain rate.

Figure 7a–c show the three-dimensional representations of elastic modulus, yield strength, and ultimate tensile strength, respectively, as functions of temperature and strain rate. The effects of both temperature and strain rate on tensile properties can be seen simultaneously in Figure 7, providing insight on the two synergistic effects. Values of R-Square and RMSE (Root Mean Squared Error) reported in Table 1 indicate reasonable fits for tensile properties. MATLAB [40] was used for generating the 3D plots.

As shown in Figure 7, the experimental data are reasonably close to the fitted surfaces. Equation (4) can be used to estimate tensile properties in design with HDPE, for example in finite element analysis. Elastic modulus and ultimate tensile strength for compression molded HDPE with specific molding condition in [31] were estimated using this polynomial equation. The results were within a maximum 20% of the reported values in [31]. However, such differences can be expected, depending on the specific molding process and conditions used.

**Figure 8:**
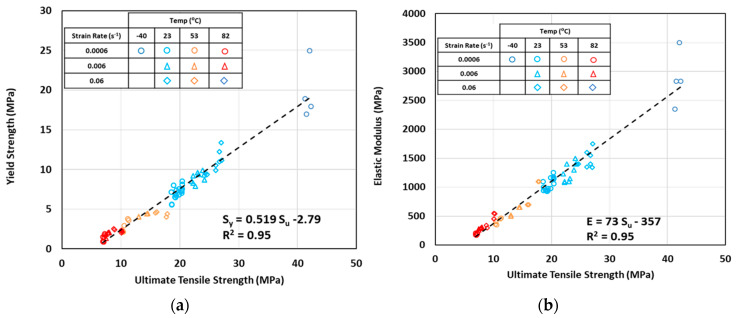
Correlations between yield strength (**a**) and elastic modulus (**b**) as linear functions of ultimate tensile strength for all temperatures and strain rates.

It was also found that yield strength and elastic modulus can be estimated as functions of ultimate tensile strength by linear functions, as shown in Figure 8a,b, respectively. Such functions were found to be independent of material (virgin, regrind, or laminated HDPE), manufacturing technique (compression molding, injection molding, or blow molding), thickness (1, 2 or 4 mm), temperature (−40, 23, 53 and 82 °C), and strain rate, as can be observed from these figures.

These linear relations for HDPE are expressed by the following equations:

(5) $$S_{y}=0.519\;S_{u}-2.79\;(\mathrm{MPa})$$

(6) $$E=73\;S_{u}-357\;(\mathrm{MPa})$$

Such linear equations can be used to estimate E and $S_{y}$ which are sometimes used in design and more difficult to obtain than the ultimate tensile strength $S_{u}$.

## 4. Conclusions

This study was designed to investigate the effects of different factors on tensile behavior of HDPE including processing technique, thickness, temperature, and strain rate. Virgin, regrind, and laminated HDPE materials were included in the experimental study. Based on the obtained experimental results and performed analysis, the following conclusions can be made:

1. No anisotropy effect on tensile properties was observed neither for compression molded nor for injection molded HDPE.
2. Processing technique and thickness affect the tensile properties of HDPE synergistically. There was no thickness effect on tensile properties for HDPE with the compression molding process, while there was a 23% increase in elastic modulus and a 21% decrease in ultimate tensile strength by increasing thickness from 2 to 4 mm in injection molding.
3. A slight improvement in elastic modulus and ultimate tensile strength was observed for HDPE after the regrinding process. However, regardless of the processing technique of virgin, regrind, and laminated HDPE, there was no significant difference observed for these three HDPE material forms with 4 mm thickness in terms of tensile properties.
4. Stress–strain curves were greatly influenced by temperature. An exponential reduction in tensile strength and elastic modulus was seen by increasing temperature regardless of the specimen thickness. Elastic modulus and ultimate tensile strength linearly increase at higher strain rates. However, strain at ultimate tensile strength is reduced as strain rate increases.
5. Polynomial functions could be fitted to all experimental data to estimate tensile properties of HDPE as functions of temperature and strain rate.
6. Yield strength and elastic modulus were correlated with ultimate tensile strength with linear functions, independent of material (virgin, regrind, or laminated HDPE), manufacturing technique (compression molding, injection molding, or blow molding), thickness (1, 2 or 4 mm), temperature (−40, 23, 53 and 82 °C), and strain rate.

In summary, there is much global interest in HDPE because of its application in a broad range of industries by using a variety of molding techniques. Components and structures made of this material are subjected to a variety of loadings and environments, therefore, the importance of the effects of strain rate and temperature. The findings in this study can facilitate improved design and prediction of mechanical performance of such components and structures made of HDPE.

## Figures and Tables

**Table 1:** Constants of empirical relationships for HDPE tensile properties.

Property	Symbol	A	B	C	D	$R^{2}$	RMSE
Elastic Modulus, MPa	E	0.11	−27.7	192.4	2253	0.94	157
Ultimate Tensile Strength, MPa	$S_{u}$	8.75 × 10^−4^	−0.341	2.68	36.0	0.98	1.06
Tensile Yield Strength, MPa	$S_{y}$	6.65 × 10^−4^	−0.189	1.22	15.4	0.94	1.18
